# Supplementary material for: Resolving the full spectrum of human genome variation using Linked-Reads
Source: Genome Res. 2019 Apr;29(4):635–45. doi: 10.1101/gr.234443.118 (PMC6442396; doi:10.1101/gr.234443.118)
Supplement: Supplemental Material [file supp_gr.234443.118_Supplemental_longranger-2.2.zip › longranger-2.2.tar.bz2!/lr2.2-public/lib/stan/cmdstan/stan/doc/api/html/namespacemembers_l.html]

Stan: Namespace Members


|  |  |
| --- | --- |
|  | Stan  2.5.0  probability, sampling & optimization |


- Main Page
- Related Pages
- Namespaces
- Classes
- Files

- Namespace List
- Namespace Members

- All
- Functions
- Variables
- Typedefs
- Enumerations
- Enumerator

- a
- b
- c
- d
- e
- f
- g
- h
- i
- j
- l
- m
- n
- o
- p
- q
- r
- s
- t
- u
- v
- w


All Classes Namespaces Files Functions Variables Typedefs Enumerations Enumerator Friends Macros Pages

Here is a list of all namespace members with links to the namespace documentation for each member:

### - l -

- lb\_constrain()
  : stan::prob
- lb\_free()
  : stan::prob
- lbeta()
  : stan::agrad
  , stan::math
- left\_division\_f
  : stan::gm
- length()
  : stan
- length\_mvt()
  : stan
- lgamma()
  : stan::agrad
  , stan::math
- lkj\_corr\_cholesky\_log()
  : stan::prob
- lkj\_corr\_cholesky\_rng()
  : stan::prob
- lkj\_corr\_log()
  : stan::prob
- lkj\_corr\_rng()
  : stan::prob
- lkj\_cov\_log()
  : stan::prob
- lmgamma()
  : stan::agrad
  , stan::math
- local\_origin
  : stan::gm
- log()
  : stan::agrad
  , stan::math
- log10()
  : stan::agrad
  , stan::math
- log1m()
  : stan::agrad
  , stan::math
- log1m\_exp()
  : stan::agrad
  , stan::math
- log1m\_inv\_logit()
  : stan::agrad
  , stan::math
- log1p()
  : stan::agrad
  , stan::math
- log1p\_exp()
  : stan::agrad
  , stan::math
- log2()
  : stan::agrad
  , stan::math
- LOG\_10
  : stan::math
- LOG\_2
  : stan::math
- log\_determinant()
  : stan::agrad
  , stan::math
- log\_determinant\_ldlt()
  : stan::agrad
  , stan::math
- log\_determinant\_spd()
  : stan::agrad
  , stan::math
- log\_diff\_exp()
  : stan::agrad
  , stan::math
- log\_falling\_factorial()
  : stan::agrad
  , stan::math
- log\_inv\_logit()
  : stan::agrad
  , stan::math
- log\_inv\_logit\_diff()
  : stan::prob
- LOG\_PI\_OVER\_FOUR
  : stan::math
- log\_prob\_grad()
  : stan::model
- log\_prob\_propto()
  : stan::model
- log\_rising\_factorial()
  : stan::agrad
  , stan::math
- log\_softmax()
  : stan::agrad
  , stan::math
- log\_sum\_exp()
  : stan::agrad
  , stan::math
- logical\_and()
  : stan::math
- logical\_eq()
  : stan::math
- logical\_gt()
  : stan::math
- logical\_gte()
  : stan::math
- logical\_lt()
  : stan::math
- logical\_lte()
  : stan::math
- logical\_negate\_expr\_f
  : stan::gm
- logical\_negation()
  : stan::math
- logical\_neq()
  : stan::math
- logical\_or()
  : stan::math
- logistic\_ccdf\_log()
  : stan::prob
- logistic\_cdf()
  : stan::prob
- logistic\_cdf\_log()
  : stan::prob
- logistic\_log()
  : stan::prob
- logistic\_rng()
  : stan::prob
- logit()
  : stan::agrad
  , stan::math
- lognormal\_ccdf\_log()
  : stan::prob
- lognormal\_cdf()
  : stan::prob
- lognormal\_cdf\_log()
  : stan::prob
- lognormal\_log()
  : stan::prob
- lognormal\_rng()
  : stan::prob
- lp\_no\_jacobian()
  : stan::optimization
- lub\_constrain()
  : stan::prob
- lub\_free()
  : stan::prob


---

[ Stan Home Page ]

*© 2011–2014,
Stan Development Team.*
